# Main findings and advances in biomedical engineering and bioinformatics from IWBBIO 2015

**DOI:** 10.1186/s12938-016-0187-9

**Published:** 2016-07-15

**Authors:** Franscisco M. Ortuño, Olga Valenzuela, Peter Glösekötter, Ignacio Rojas

**Affiliations:** 1 Department of Computer Architecture and Computer Technology, CITIC-UGR, University of Granada, 18071 Granada, Spain; 2 Department of Applied Mathematics, University of Granada, 18071 Granada, Spain; 3 Department of Electrical Engineering and Computer Sciences, Muenster University of Applied Sciences, 48565 Steinfurt, Germany

In the current supplement, we are proud to present ten relevant contributions from the 3rd International Work-Conference on Bioinformatics and Biomedical Engineering (IWBBIO 2015), which was held during April 15–17, 2015 in Granada (Spain) [1, 2]. These contributions have been chosen because of their quality and the importance of their findings.

IWBBIO 2015 aimed to follow the same friendly environment as previous editions, allowing researchers, scientists and students to show their latest ideas, discoveries and outcomes. The conference sought to focus on diverse fields to create multidisciplinary researches integrating areas like biomedical engineering, computer science, mathematics, artificial intelligence, bioinformatics, statistics or biomedicine. IWBBIO 2015 also promoted the meeting, cooperation and collaboration of scientists with the presentation and discussion of their latest relevant ideas and work-in-progress. These ideas provided important advances to the scientific community in fields like genomics, next-generation sequencing, drug design and advanced pharmacology, biomedical modelling and e-health, among other. Additionally, IWBBIO 2015 was honored with the presence of three invited plenary speakers: Prof. Alfonso Valencia, Prof. Patrick Aloy and Prof. Xavier Estivill. These plenary lectures strengthened the aim of this conference for the diffusion and the discussion of high quality researches from some of the most recognized scientists in these fields.

The IWBBIO 2015 has continued as a two-track conference, increasing the number of sessions to a total of 24 oral and 2 poster sessions. It received more than 210 contributions which were reviewed by at least two referees from our estimated program and steering committees. The conference continues accepting both full and abstract submissions for presentations. However, it still maintained a high rate of full contributions against abstracts. IWBBIO 2015 received more than 200 attendees from diverse European nationalities (Spain, United Kingdom, France, Italy, Poland, etc) but also overseas countries like United Stated, Korea, China or India.

Those contributions which were considered more relevant taking into account the evaluation and opinion of reviewers and chairmen were then invited to participate in this supplement for the BioMedical Engineering OnLine journal. In this regard, this supplement is specially based on particular technological implementations and developments oriented to facilitate and improve healthcare and wellness. All the novel researches showed in this supplement have a massively interdisciplinary background, dealing with hot topics in the area of biomedicine and biomedical engineering like e-health, diagnosis or biology systems but also statistics and computer science like cloud computing, big data or mathematical modelling.

Thus, the first paper authored by Gimenez et al. [3] describes a mathematical implementation of non-uniform flow models to simulate tilted holes and conical holes in the obstruction of ventricular catheters. Although these simulations have been developed in an ideal scenario, they provide promising results about the consequences of variations in diameters as well as tilt angles of the holes. This novel design improves the disadvantage of having too small holes which appear in other commercially available implementations.

The article by Gamberger el al. [4] proposes a novel clustering algorithm which is able to determine small and heterogeneous subpopulations in Alzheimer’s disease (AD) patients. This method, called multilayer clustering, defines each clinical or biological descriptor as a “layer”, building clusters based on the co-existence of properties. This methodology was evaluated with a dataset with 33 properties and 916 patients with significant problems with dementia. Even though the methodology should be validated in other domains, it has shown the successful identification of subpopulations in the AD domain.

Abbasi et al. [5] address in their article one traditional but still challenging problem in bioinformatics, the multiple sequence alignment (MSA). In this case, authors introduce a novel heuristic solution based on local search and an optimization based on the number of indels and a substitution score (multiobjective approach). From these bases, several implementations and configurations are proposed and compared including perturbation techniques to avoid suboptimal solutions due to local maximums. Taking into consideration the outcomes, this study has shown a significant improvement in alignments in a reasonable time.

Following, the paper by Molina-Recio et al. [6] analyzes the effect of multidisciplinary research when developing mobile applications based on eHealth (also called mHealth). This systematic review highlights the exponential increase of research publications in this area and the importance of interdisciplinary teams involving more health professionals, whose contribution is currently lacking from. Nevertheless, authors of this study also conclude that the impact in the analyzed publications could be not necessarily correlated with the presence of multidisciplinary teams.

In the next article, Macdonald et al. [7] presents an adaptive gradient matching approach based on Gaussian processes to estimate parameters in biological pathways, which are habitually expressed as pairs of ordinary differential equations (ODEs). This contribution is mainly based on a mathematical and numerical modelling but with a clearly promising application in the inference of system biology parameters. In fact, according to the provided results, the proposed approach outperforms other similar solutions when estimating parameters in two benchmarks to describe the voltage potential across the cell membrane and the protein signaling transduction pathway, respectively.

The paper by Cisar et al. [8] describes BioWes, a complete and well-designed repository to store and to manage experimental descriptors and data. This tool is specifically designed taking into account the importance of “Big Data” and how dealing with it. With this purpose, it includes implementations for desktop application, web interface and web-based interface mobile devices. BioWes has been validated in the context of the AQUAEXCEL international infrastructure project in order to organize and store data from aquaculture experiments.

The next contribution by Ortega et al. [9] analyzes different feature selection techniques in multi-resolution analysis (MRA) to classify electroencephalographic (EEG) images for brain-computer interfaces (BCIs). Several feature selection approaches are compared based on evolutionary multi-objective techniques and different structures of classifiers. Authors statistically probe that the proposed feature selection algorithms outperform the baseline MRA in terms of computational costs given the reduction in the number of features, providing similar or better classification performances.

Subsequently, Baños et al. [10] present in their publication a system for personalized medicine, healthcare and wellness based on a new digital framework called Mining Minds. The proposed system exploits the advantages of cloud computing storage and high performance computing but with the novelty of incorporating wearable technology and Big Data. This framework addresses an efficient, flexible and robust design which makes it a promising and potential tool for other clinical developments and applications.

The article from Larriba et al. [11] proposes a novel baby dinosaur robotic pet, called Pleo, to reduce pain and anxiety in hospitalized children. Simultaneously, this therapeutic pet is wireless connected to assist clinical personal to monitor and understand the child behavior through an Android app. This article describes the whole architecture of Pleo robot in detail but it also shows the successful results obtained from preliminary tests with children in hospital.

Finally, Soria-Morillo et al. [12] describe a novel discrete classification algorithm to determine human reactions on TV advertisements from low-cost EEG signals. This algorithm is presented by integrating signal collection/transformation stages with different supervised machine learning approaches like C4.5, ANN or an own-designed discretization procedure. After validating and comparing these classifier’s performances, authors showed their approach clearly outperforms other tools in terms of accuracy and power consumption.

As Guest editors, we would like to express our thankfulness to all the authors contributing with their high quality researches to the achievement of this supplement. Also, we are very grateful to expert scientists that have actively collaborated with their recommendations and suggestions to review and improve these contributions. We would like to sincerely acknowledge Profs. Kenneth R. Foster and Fong-Chin Su, Editors-in-Chief of BioMedical Engineering Online, for giving again the opportunity to publish this supplement in this relevant journal. We specially thank to Mr. Omar El Bakry for his excellent and constant support with the publication and edition of this supplement. It has been an honor for us to participate in it. We finally invite authors and readers of this supplement to submit their recent works to future editions of IWBBIO, which will be announced at http://iwbbio.ugr.es.

## References

[1] Ortuño FM, Rojas I (eds) (2015) Bioinformatics and biomedical engineering—third international conference, IWBBIO 2015. In: Proceedings Part I. Lecture notes in computer science, vol 9043

[2] Ortuño FM, Rojas I (eds) (2015) Bioinformatics and biomedical engineering—third international conference, IWBBIO 2015. In: Proceedings part II. Lecture notes in computer science, vol 9044

[3] Gimenez A, Galarza M, Pellicer O, Valero J, Amigo JM (2016). Influence of the hole geometry on the flow distribution in ventricular catheters for hydrocephalus. Biomed Eng Online.

[4] Gamberger D, Zenko B, Mitelpunkt A, Lavra N (2016). The Alzheimer’s disease neuroimaging-initiative: homogeneous clusters of Alzheimer’s disease patient population. Biomed Eng Online.

[5] Abbasi M, Paquete L, Pereira FB (2016). Heuristics for multiobjective multiple sequence alignment. Biomed Eng Online.

[6] Molina-Recio G, García-Hernández L, Molina-Luque R, Salas-Morera L (2016). The role of interdisciplinary research team in the impact of health apps in health and computer science publications: a systematic review. Biomed Eng Online.

[7] Macdonald B, Niu M, Rogers S, Filippone M, Husmeier D (2016). Approximate parameter inference in systems biology using gradient matching: a comparative evaluation. Biomed Eng Online.

[8] Cisar P, Soloviov D, Barta A, Urban J, Stys D (2016). BioWes—from design of experiment, through protocol to repository, control, standardization and back-tracking. Biomed Eng Online.

[9] Ortega J, Asensio-Cubero J, Gan JQ, Ortiz A (2016). Classification of motor imagery tasks for BCI with multiresolution analysis and multiobjective feature selection. Biomed Eng Online.

[10] Banos O, Amin MB, Khan WA, Afzal M, Hussain M, Kang BH, Lee S (2016). The mining minds digital health and wellness framework. Biomed Eng Online.

[11] Larriba F, Raya C, Angulo C, Albo-Canals J, Díaz M, Boldú R (2016). Externalising moods and psychological states in a cloud based system to enhance a pet-robot and child’s interaction. Biomed Eng Online.

[12] Soria-Morillo LM, Alvarez-Garcia JA, Gonzalez-Abril L, Ortega JA (2016). Discrete classification technique applied to TV advertisements liking recognition system based on low-cost EEG headsets. Biomed Eng Online.

